# »Ich bin seit Montag Zuhause in Quarantäne« – Zur Verbindung von Erzählen und Argumentieren in Social-Media-Kommentaren zu politischen Reden

**DOI:** 10.1007/s41244-021-00199-y

**Published:** 2021-05-06

**Authors:** Sascha Michel, Daniel Pfurtscheller

**Affiliations:** 1 Institut für Sprach- und Kommunikationswissenschaft, RWTH Aachen, Aachen, Deutschland; 2 Institut für Germanistik, Universität Wien, Wien, Österreich

**Keywords:** Erzählen, Small Stories, Narration, Argumentation, Politische Kommunikation, Medienaneignung, Covid-19, Facebook, YouTube, Instagram, Twitter, Narration, Small Stories, Argumentation, Political Communication, Media Appropriation, Covid-19, Facebook, YouTube, Instagram, Twitter

## Abstract

Dieser Artikel untersucht die Verbindung von Erzählen und Argumentieren im Kontext politischer Reden zur Coronakrise. Die qualitative Untersuchung basiert auf einem Korpus von 650 Kommentaren, die Nutzer*innen im Anschluss an die sogenannten Lockdown-Reden von Sebastian Kurz und Angela Merkel im März 2020 auf verschiedenen Social-Media-Plattformen veröffentlicht haben. In den Kommentaren lassen sich Formen des fragmentarischen Erzählens nachvollziehen, das zur Stützung von Argumenten und zur Positionierung im kommunikativen Gefüge der beteiligten Akteure im Kontext der verkündeten Lockdown-Maßnahmen dient.

## Abstract (en)

This article examines narrative practices in the context of political speeches on the corona crisis. The qualitative analysis is based on a corpus of 650 social media comments posted in response to the so-called lockdown speeches by Sebastian Kurz and Angela Merkel in March 2020. The comments feature forms of fragmentary narration that are used by the participants both as resources of credibility and descriptiveness and as a means of positioning themselves in the context of the lockdown measures being announced.

## Einleitung

Das vom Institut für Deutsche Sprache herausgegebene *Neologismenwörterbuch zur Coronakrise *bestimmt *Lockdown* als »Zeitraum, in dem fast alle wirtschaftlichen und gesellschaftlichen Aktivitäten auf politische Anordnung hin stillgelegt sind« (Neologismenwörterbuch 2020). Der weltweite Ausbruch der Atemwegserkrankung COVID-19 im Frühjahr 2020 war für viele Menschen der erste Kontakt mit der bisher unbekannten Situation einer behördlich angeordneten Massenquarantäne und dem völligen Stillstand des öffentlichen Lebens. In Österreich und in Deutschland waren die ersten strengen Ausgangsbeschränkungen dieser Art im März 2020 in Kraft. In diesem Beitrag fokussieren wir die Ankündigung dieser Lockdown-Maßnahmen durch die Regierungsspitzen in beiden Ländern und untersuchen anhand von Social-Media-Kommentaren, welche Rolle das alltägliche Erzählen hinsichtlich der interaktiven Nachbearbeitung und argumentativen Aneignung der in den Lockdown-Reden verkündeten Maßnahmen spielt.^1^

Die Argumentationsforschung hat sich in ihrer bis auf die antike Rhetorik zurückverfolgbaren Geschichte immer wieder mit der Frage nach der Rolle des Erzählens für das Argumentieren befasst (für einen Überblick vgl. Schwarze 2019; Hannken-Illjes 2019). Dass Erzählen eine fundamentale Rolle »im Leben des Einzelnen und im kommunikativen Haushalt einer Gesellschaft spielt« (Fritz 2017, S. 429) und in der Regel kein Selbstzweck ist, sondern etwas, mit dem etwas getan wird und das an bestimmte (argumentative) Funktionen gekoppelt ist, gilt gleichermaßen für das digitale Erzählen (Tophinke 2017). Aus medienlinguistischer Perspektive stellt sich dabei die Frage, wie durch digitale Medienumgebungen bestehende Praktiken verändert und mit anderen Aktivitäten verknüpft werden (z. B. dem Teilen und Kommentieren von massenmedialen Inhalten). Kommentare auf Social-Media-Plattformen, wo sich private Nachrichten, humorvolle Memes und politische Schlagzeilen zu einer algorithmisch kuratierten Melange vermengen, müssen in der Regel gleichzeitig mehrere Adressatengruppen ansprechen. Die linguistische Forschung zum digitalen Erzählen hat sich in diesem Zusammenhang damit befasst, wie Erzählen und insbesondere Praktiken der narrativen (Selbst‑)Positionierung (*narrative stance*, Georgakopoulou 2016b) helfen, um mit diesem *context collapse* (Marwick/Boyd 2011) digitaler Medienumgebungen umzugehen.

In unserer qualitativen Studie untersuchen wir öffentliche Stellungnahmen von Nutzer*innen, die im Anschluss an die Verbreitung der Lockdown-Reden in Österreich und Deutschland im März 2020 auf mehreren verschiedenen Social-Media-Plattformen geschrieben wurden. Social-Media-Kommentare sind stark kontextgebundene Textbeiträge und zeichnen sich in der Regel durch fragmentarische Formen des Erzählens und Argumentierens aus. Gerade durch diese Verkürzungen erscheinen die Anschlusskommentare als geeignetes Material, um politisch motivierten Erzählungen (Weidacher 2018) und argumentativen Funktionen gemeinschaftlich geteilter Narrative auf die Spur zu kommen. Unter Narrativen verstehen wir »kulturelle Orientierungsfolien für individuelles Handeln und Erleben« (Bubenhofer/Müller/Scharloth 2013, S. 423), die als diskursiv entstandene Muster empirisch auffindbar sind (vgl. Bubenhofer 2018, S. 373).

Im Folgenden werden wir nach einigen allgemeinen Ausführungen zum Verhältnis von Erzählen und Argumentieren in der Social-Media-Interaktion (Kap. 2) anhand eines Korpus von Social-Media-Daten zu politischen Reden (Kap. 3) induktiv herausarbeiten, wie sich alltägliches Erzählen und Argumentieren in Social-Media-Kommentaren zu politischen Reden verbindet. Dabei wird sich zeigen, dass Erzählen neben dem sprachlichen Ausdruck von Alltagserlebnissen auch zur Stützung von Argumenten und zur Positionierung im kommunikativen Gefüge der beteiligten Akteure dient (Kap. 4). Um die Rolle zu beleuchten, die das fragmentarische Erzählen für das Argumentieren in der interaktiven Nachbearbeitung von politischen Reden auf Social-Media-Plattformen hat, fragen wir danach, welche Anschlussstellen für erzählendes Argumentieren es in den Reden gibt, wie sich Miniatur-Erzählungen in den jeweiligen kommunikativen Konstellationen und medialen Umgebungen der untersuchten Textgattungen jeweils ausprägen und welche spezifischen argumentativen Funktionen sie erfüllen. Schließlich fassen wir die wichtigsten Beobachtungen zusammen und formulieren einen Ausblick (Kap. 5).

## Erzählen und Argumentieren in der (Social-Media‑)Interaktion

Aus linguistischer Sicht ist Erzählen eine kommunikative Praktik, die im Alltag für unterschiedliche Aufgaben eingesetzt werden kann und damit selten alleine steht: »Erzählen ist als sprachliches Handeln integriert in die sonstigen Handlungsbezüge der gesellschaftlichen Aktanten« (Ehlich 1980, S. 20). Im Alltag werden Geschichten in der Regel erzählt, um etwas zu tun (Schegloff 1997, S. 97): sich zu beschweren oder zu entschuldigen, anderen etwas mitzuteilen oder von etwas zu überzeugen etc. Mit Erzählen können wir im Alltag Zugehörigkeit markieren, soziale Nähe herstellen, Weltsichten teilen und wechselseitig bestätigen, aber auch Wissen aufbauen, organisieren und verarbeiten (vgl. Tophinke 2009 S. 257 f.; Spieß/Tophinke 2018, S. 195). Alltägliches Erzählen ist somit kein Selbstzweck, sondern mit bestimmten kommunikativen Absichten verbunden oder darauf ausgerichtet, eine Umgebung zu etablieren, in dessen Verlauf oder Kontext weitere kommunikative Handlungen vollzogen werden können.

Aus diesem Grund ist alltägliches Erzählen auch für argumentative Zusammenhänge relevant. So hat etwa Schwarze (2019) in ihrer gesprächslinguistischen Studie hervorgehoben, dass Erzählen in argumentativen Zusammenhängen mehrere Funktionen erfüllen kann: »[P]rominent sind Anschaulichkeitsherstellung, Wissensgenerierung und Überzeugung. Durch die Nutzung der Erfahrungsbasiertheit wird mehr Anschaulichkeit, Sinnlichkeit, persönliche Perspektive und Konkretheit beim Argumentieren hergestellt, zudem ist die Erlebnisperspektive eine Glaubwürdigkeitsressource.« (Schwarze 2019, S. 67–68) Argumentativ-funktionalisiertes Erzählen ist dabei nicht intrinsisch motiviert (*autotelic mode*, Ryan 2006, S. 13), sondern zweckgerichtet (*utilitarian mode*, Ryan 2006, S. 13). Beispielerzählungen, die sich im Rahmen einer impliziten Beweisführung als Überzeugungsmittel (im Sinne Aristoteles) nutzen lassen, werden so auch in der Politik zum Argumentieren verwendet. Die Funktionalisierung als »exemplarische Geschicht[e]« (Keppler 1988, S. 39) kann als argumentative Strategie gesehen werden, mit der sich ein moralischer Appell absichern lässt: »Beispiele werden häufig da erzählt, wo es um Erkenntnis von Menschen und Situationen, um Fragen der Moral und praktischen Lebensführung geht« (Retting 2014, S. 135). Girnth/Burggraf (2019) sowie Klein (2019) legen dar, dass im politischen Sprachgebrauch Narrationen vor allem der Realisierung bestimmter Argumentationstopoi dienen: »Der narratio kommt dann eine besondere Funktion innerhalb des Daten- und Validationstopos zu, da sie ein effektives Mittel der Schilderung der Situationsdaten einschließlich ihrer Bewertung darstellt« (Girnth/Burggraf 2019, S. 568). Häufig lassen sich Erzählungen demnach als Exemplum verstehen, die der Ratio von Argumenten eine emotionale Ebene hinzufügen und so die persuasive Wirkmacht politischer Rhetorik erhöhen (vgl. Girnth/Burggraf 2019, S. 568 f, zum Beispielbeweis als alltäglichem Argumentationsschema vgl. Kienpointner 1992).

Aus dieser Perspektive, die nach dem argumentativen Gehalt von alltäglichen Erzählungen fragt, befassen wir uns in der vorliegenden Studie mit digitalen Erzählpraktiken. Das internetbasierte Erzählen ist von der Forschung in letzter Zeit vermehrt in den Blick genommen worden (einen Überblick für den Bereich des Erzählens im Internet gibt Tophinke 2017, siehe auch Simanowski 2017; Page 2018). Zu den gesprächs- und textlinguistischen Merkmalen des Erzählens treten hier medienbezogene Besonderheiten. Wie Oloff/König (2018) am Beispiel einer Fernsehunterhaltungssendung zeigen, wird die Art und Weise des multimodalen Erzählens maßgeblich durch das Dispositiv des Mediums – hier: des Fernsehens – geprägt.^2^ Diese dispositive Prägung – Luginbühl (2019, S. 125) spricht von einer »medialen Durchformung« – lässt sich auch mit Bezug auf internetbasierte Medien beobachten. Intensiv erforscht wurde der Bereich der sogenannten »Small Stories« (Bamberg 2006; Georgakopoulou 2007). Dieser Sammelbegriff, der eine Vielzahl von in der klassischen Erzählforschung weniger beachteten narrativen Aktivitäten abdeckt (Georgakopoulou 2006, S. 123), wurde ursprünglich innerhalb der Positionierungsanalyse mündlicher Interaktion entwickelt und als Analyserahmen für Social-Media-Interaktion ausgebaut (vgl. Bamberg/Georgakopoulou 2008; Günthner 2012; Georgakopoulou 2016a). Für Small Stories ist charakteristisch, dass sie meist fragmentarisch sind und die klassischen Erzählschritte des persönlichen Erzählens (*abstract – orientation – complicating action – resolution – coda – evaluation*, Labov/Waletzky 1967) nur unvollständig abdecken. Hinzu kommt, dass Social-Media-Erzählungen häufig zeitverzögert (*delayed*, Dayter 2015) und interaktiv durch Ko-Erzähler*innen konstituiert werden (Page/Harper/Frobenius 2013) sowie alltägliche Themen aufgreifen, die in der klassischen Forschung als wenig bis gar nicht »erzählwürdig« gelten: »There is also a tendency for reporting mundane, ordinary and, in some cases, trivial events from the poster’s every day life, rather than big complications or disruptions« (Georgakopoulou 2016a, S. 302). Als einfache Erzählungen, die selten ausgebaut sind, werden Small Stories als Teil der Identitätsarbeit gesehen. Beiläufige Formen des Erzählens lassen sich überall dort finden, wo sich Nutzer*innen »als Teil der identitätskonstruktiven Selbstthematisierungen und der sozialen Beziehungspflege über ihre Alltagserlebnisse austauschen, diese kommentieren und bewerten« (Tophinke 2017, S. 70).

Im Gegensatz zur mündlichen Interaktion, wo Erzählen als »multimodal hervorgebrachte Aktivität« (König/Oloff 2018, S. 215) im Raum verzögerungsfrei wahrnehmbar werden kann, gibt es in Social-Media-Umgebungen in der Regel keine körperliche Kopräsenz oder Möglichkeit einer zeitlichen Synchronisation. Hier ist Interaktion deutlich anders organisiert. In unserem Fall beruhen die Interaktionen auf dem Austausch von Social-Medial-Posts, das sind (potenziell multimodale) Textbeiträge, die en bloc verschickt werden. Mit Beißwenger (2020) kann man hier von einer textformen-basierten Interaktion sprechen, die darauf ausgelegt ist, dass sich Nutzer*innen an einem sich dynamisch entfaltenden Interaktionsgeschehen beteiligen können. Eine wichtige Organisationsebene dafür ist die flächige Sichtbarkeit der vorgängigen Aktivitäten, der Beiträge und Kommentare in einem Verlaufsprotokoll, das die Interaktion in eine lineare Ordnung bringt und für die wiederholbare Rezeption greifbar macht. Soziale Medien sind als Erzähl- und Argumentationsumgebungen *designed spaces* (Pfurtscheller 2019, 115–116), die zahlreiche Angebote und Vorgaben (*affordances* und *constraints*) hinsichtlich Nutzung und Beteiligungsmöglichkeiten machen. Generell basiert die Kommunikation in sozialen Medien auf vier Affordanzen (vgl. Weidacher 2018, S. 321 f.): 1. Hypertextualität, 2. Multimodalität, 3. Fluidität, 4. Dialogizität. Eine medienlinguistische Perspektive auf Erzählen und Argumentieren steht vor der Aufgabe, die narrative Entfaltung, die durch diese technologischen Affordanzen ermöglicht wird, vor dem Hintergrund bereits etablierter Ausdrucksformen zu untersuchen. Man könnte nach Androutsopoulos (2016) auch von mediatisierten Erzählpraktiken sprechen, die sich als Gefüge kommunikativer Handlungen bezeichnen lassen, die digitale Technologien miteinbeziehen und in ihrer Pragmatik mehr oder weniger stark auf prä-digitalen Vorläufern des Erzählens und Argumentierens beruhen. Für den Bereich des Erzählens auf Social-Media-Plattformen, der hier im Zentrum steht, kann man den Aspekt des Teilens (*sharing*) zentral setzen. In ihrer Konzeption von ›geteilten Erzählungen‹ hat Page (2018) dafür eine intertextuelle Analyseperspektive vorgeschlagen, um sowohl Mitteilungs- als auch Distributionsaspekte des vernetzten Erzählens im Blick zu behalten: »In shared stories, intertextuality takes centre stage as an aspect of the narrative which is both part of the telling (reflecting the meaning of ›sharing as telling‹) and part of the distributive processes of sharing« (Page 2018, S. 204).

Diese allgemeinen Affordanzen formen sich in einem weiteren Schritt plattformspezifisch aus. Soziale Medien entfalten als Kommunikationskanäle dabei eine dispositive Wirkung^3^: Beispielsweise ist für Facebook eine relativ flache Interaktionsstruktur typisch, die keine verzweigten Kommentarverläufe befördert. Eine interaktive Nachbearbeitung von Erzählungen ist zwar möglich, beschränkt sich aber meist auf kurze Kommentare. Nach Tophinke (2009, 2017) sind für Blogs der 1990er-Jahre Anschlusserzählungen in Form von Kommentaren mit eigenständigen *second stories*, die den Plot aufgreifen, typisch, allerdings spiele dies in sozialen Medien wie Facebook kaum eine Rolle; vielmehr würden Reaktions-Buttons und formelhafte Sprache verwendet, um Postings als erzählenswert zu bestätigen (vgl. Tophinke 2017, S. 72). Inwieweit sich solche fallspezifischen Beobachtungen des Zusammenspiels von Technologie und Erzählpraktiken über Gruppen und Social-Media-Plattformen hinweg beobachten lassen, ist unklar. Um funktionale sowie (medien-) kulturspezifische Unterschiede in den medialen Ausprägungen erkennen zu können, sind daher insbesondere plattformübergreifende Analysen digitalen Erzählens gefragt.

Unsere Studie betrachtet alltägliches Erzählen und Argumentieren im Kontext politischer Reden und ihrer mediatisierten Anschlusskommunikation auf mehreren unterschiedlichen Social-Media-Diensten. Einerseits lassen sich politische Reden selbst hinsichtlich vorfindlicher Formen des argumentativen Erzählens bzw. erzählender Argumentation untersuchen (vgl. Hannken-Illjes 2019; vgl. Weidacher 2018). Andererseits werden die Reden selbst in mediatisierter Form auf Social-Media präsentiert und von den Nutzer*innen kommentiert. Während sich diese Kommunikatperspektive auf die Analyse von Narration und Argumentation mit Bezug auf digitale politische Texte gerade erst am Anfang befindet, so gilt dies erst recht für die Aneignungsebene, also für die Analyse von Online-Reaktionen, die traditionell eher weniger im Zentrum politolinguistischer Untersuchungen steht. Für diese Ebene ist charakteristisch, dass sich Nutzer*innen über Medienkommunikate äußern und austauschen, was wiederum Rückschlüsse darauf zulässt, wie sie sich die Kommunikatinhalte aneignen, d.h. auf welche Themen sie wie referieren und welche Sprachhandlungen dabei in Erscheinung treten. Für das fernsehbegleitende Twittern etwa, dem sogenannten Social TV, ist kennzeichnend, dass es eher um die Selbstdarstellung von Nutzer*innen geht als um die themen- und diskursbezogene Aushandlung bzw. Argumentation (vgl. Klemm/Michel 2014a). Obgleich sich die Aneignungshandlungen kaum vom fernsehbegleitenden Sprechen in intimer und geschützter Atmosphäre der familiären Kleingruppe unterscheiden, so zeigen formale Eigenschaften wie die Verwendung von Hashtags sowie die im Vergleich zur Mündlichkeit ausgeprägte Elaboriertheit der Äußerungen, dass fernsehbegleitende Tweets vor allem der Selbstinszenierung vor einem öffentlichen Publikum dienen. Alltägliche Formen der Medienaneignung sind stark expressiv, dennoch werden Tweets und die Kommentarfunktionen diverser Plattformen im Alltag durchaus auch genutzt, um (begründete) Kritik an Medienkommunikaten zu äußern. Auch wenn ausgebaute Argumentationen selten sind, stellt sich die Frage, inwieweit sich in diesen Miniaturformen der kommunikativen Medienaneignung Spuren eines »kritischen Publikums« (Pfurtscheller 2020, S. 269) finden lassen. Während die thematischen Bezugnahmen sowie die Aneignungshandlungen von fernsehbegleitender digitaler Anschlusskommunikation relativ gut erforscht sind (vgl. z. B. Göttlich et al. 2017; Klemm/Michel 2014a, 2014b, 2016; Schneider/Buschow 2019), zeigt sich hier mit Blick auf Narration und dem Wechselspiel zwischen Narration und Argumentation – vor allem hinsichtlich fernsehvermittelter politischer Reden – ein Desiderat.

Wir wollen dieses Desiderat im Folgenden aufgreifen und die Anschlusskommunikation zu den sogenannten *Lockdown*-Reden von Angela Merkel und Sebastian Kurz in der Frühphase der Coronakrise an einem kleinen Ausschnitt analysieren. Damit knüpfen wir an einen aktuellen und zugleich transkulturellen Diskurs an, der eine vergleichende Analyse ermöglicht. Als Untersuchungsgegenstand greifen wir auf Nutzer*innenkommentare auf den Plattformen YouTube, Twitter, Facebook und Instagram zurück, um einen möglichst differenzierten, symptomatischen Einblick zu bekommen. Die folgenden Leitfragen sollen dabei schlaglichtartig beleuchtet werden:

- Welche Stellen der Corona-Ansprachen elizitieren fragmentarische Formen des Erzählens und Argumentierens?
- Wie wird fragmentarisches Erzählen und Argumentieren in den Anschlusskommentaren verbunden und welche funktionalen Muster lassen sich erkennen?
- Inwiefern werden in den Anschlusskommentaren *shared stories* (Page 2018) erzählt und welche argumentativen Funktionen haben interaktive Ko-Erzählungen?
- Welche Gemeinsamkeiten und Unterschiede in der Verbindung von Erzählen und Argumentieren zeigen sich im Vergleich der verschiedenen Social-Media-Plattformen sowie im transkulturellen Vergleich Deutschland und Österreich?

## Untersuchungsgegenstand, Datengrundlage und Vorgehen

Gegenstand unserer Studie sind Nutzer*innenkommentare, die im Anschluss an zwei Corona-Ansprachen der deutschen Bundeskanzlerin Angela Merkel und des österreichischen Bundeskanzlers Sebastian Kurz veröffentlicht wurden. Um die Vergleichbarkeit im Untersuchungsmaterial abzusichern, haben wir aus der Fülle von Reden und Pressestatements, die von beiden Regierungsspitzen Mitte März 2020 veröffentlicht wurden, zwei Ansprachen ausgewählt (im Folgenden: *Lockdown-Reden*). Beide Reden wurden in zeitlicher Nähe im Fernsehen ausgestrahlt (15. bzw. 18. März 2020), in mediatisierter Form (z. B. als Videoclips) auf unterschiedlichen Social-Media-Kanälen (z. B. von Regierungsstellen und Nachrichtenmedien) verbreitet und ähneln sich thematisch-funktional sehr. Die gedruckten Veröffentlichungen der Lockdown-Reden bilden den Prätext für die analysierten Nutzer*innenkommentare. Im Folgenden erläutern wir zunächst die Datengrundlage und gehen dann ausführlicher auf den Kontext der Untersuchung und die beiden Lockdown-Reden ein.

Zur Datenerhebung haben wir im Sinne der Methode der virtuellen Ethnografie (vgl. Bachmann/Wittel 2006) auf vier großen Social-Media-Plattformen (Facebook, YouTube, Instagram und Twitter) nach Postings zu den Lockdown-Reden gesucht und die Postings sowie die Nutzer*innenkommentare in einem On-Screen-Verfahren^4^ gesammelt. Insgesamt besteht unser Korpus aus rund 650 Nutzer*innenkommentaren, die wir im Kontext der Lockdown-Reden qualitativ ausgewertet haben. Die untersuchten Social-Media-Daten ähneln sich strukturell und funktional, sind aber in vielerlei Hinsicht unterschiedlich. Strukturell ähneln sich die Nutzer*innenkommentare, weil sie als Reaktionen auf einen übergeordneten Beitrag (ein Facebook- bzw. Instagram-Post oder YouTube-Video) im Angebot angeordnet sind. Funktional ähneln sie sich darin, dass sie die vorausgehenden Reden von Kurz bzw. Merkel kommentieren. Ähnliches gilt auch für Beiträge auf Twitter. Da es hier (abseits der Reply-Funktion) keine Kommentare gibt, muss die gemeinsame Referenz metakommunikativ abgesichert werden. Deshalb wurden die Anschlussbeiträge an die Reden über eine zeitlich eingegrenzte Hashtag- bzw. Schlagwort-Suche (Merkel, Kurz, Corona) erhoben.

Die Lockdown-Reden und die an sie anschließenden Nutzer*innenkommentare stammen aus der ersten Hochphase der Covid-19-Pandemie in Österreich und Deutschland, die medial von zahlreichen Pressekonferenzen, Sondersendungen und Eilmeldungen begleitet wurde (vgl. Gräf/Henning 2020).

In der Rede von Sebastian Kurz, die am 15. März ausgestrahlt wurde und grob gesagt aus vier Teilen besteht, wird (1.) der Ernst der Lage betont, (2.) über die getroffenen Maßnahmen berichtet, (3.) allen Parteien und ausgewählten Personen gedankt und (4.) das Publikum für die kommenden Wochen auf das erwünschte Verhalten eingeschworen. Ähnlich wie bei der Merkel-Rede dominieren Mahn- und Appellhandlungen (»Bleiben Sie zu Hause«). Die Rede hat aber auch stark emotionalisierende Züge, die sich einerseits stilistisch festmachen lassen (z. B. an expressiven Ausdrücken wie *unglaublicher Härte, massive Einschränkungen*, etc.), andererseits mit der expliziten Thematisierung von Leid, Tod und Angst manifest werden.

Für die Rede von Angela Merkel, die am 18. März ausgestrahlt wurde, hat Spieß (2020) bereits eine Analyse vorgelegt, an die unsere Untersuchung der Anschlusskommentare anknüpfen kann. Hiernach stützen sich die von Merkel hervorgebrachten Mahn‑, Appell- und Forderungshandlungen auf Situationsbewertungen, Gefahrenbenennungen und Dankesbekundungen (vgl. Spieß 2020, S. 207). Das aufgeworfene Spannungsverhältnis zwischen Individuum einerseits und Gesellschaft/Gemeinschaft andererseits manifestiert sich darüber hinaus in bestimmten lexikalischen Einheiten mit Solidaritätsfunktion (z. B. *wir, jede und jeder, gemeinsames Handeln*). Daran schließt auch das Wechselspiel zwischen Appell- und kommissiven Sprechhandlungen an, das Merkel vornimmt (vgl. Spieß 2020, S. 208). Insgesamt lässt sich die Rede in folgende Teile gliedern: (1.) Anknüpfen an Alltagsprobleme und -sorgen der Menschen, (2.) Informationen zum medizinischen Stand der Corona-Pandemie, (3.) gesellschaftspolitische Maßnahmen und Folgen zur Eindämmung.

Ebenso wie die Lockdown-Rede von Kurz weist auch Merkels Rede durchwegs emotionalisierende Züge auf, z. B. durch die Verwendung affektiver Lexeme (»Ihre Liebsten«), exemplarischer narrativer Sequenzen, die dazu dienen, den ›Helden‹ der Pandemie ein Gesicht zu geben (»[...] die als Ärzte oder Ärztinnen, im Pflegedienst oder einer in einer sonstigen Funktion in unseren Krankenhäusern und überhaupt im Gesundheitswesen arbeiten. Sie stehen für uns in diesem Kampf an der vordersten Linie. Sie sehen als Erste die Kranken und wie schwer manche Verläufe der Infektion sind. Und jeden Tag gehen Sie auf’s Neue an Ihre Arbeit und sind für die Menschen da. Was Sie leisten, ist gewaltig [...]«), Betonungen von Wörtern zwecks Hervorhebung von expressiven oder deontischen Aspekten (so im Satz: »Im Moment ist nur Abstand Ausdruck von Fürsorge«), Metaphern wie die der Personifizierung des Virus (»[...] das Virus auf seinem Weg durch Deutschland [...]«) sowie dem Dringlichkeitstopos (»Es ist ernst«, »dynamische Situation«).

Nicht zuletzt aufgrund solcher emotionalisierender Sequenzen^5^ erscheinen beide Lockdown-Reden für narrativ-argumentative Anschlusskommentare prädestiniert zu sein. Solche »Emotionalisierungstechniken« können nämlich die »Sympathiebildung und die Identifikation der Zuhörer gegenüber dem Protagonisten erhöhen« (Girnth/Burggraf 2019, S. 573), aber auch der Abgrenzung von und Kritik an der redenden Person Vorschub leisten. In jedem Fall lässt ein emotionales »Involvement« seitens der Rezipient*innen »die kognitiv-kritische Verarbeitung der argumentativen Inhalte« (Girnth/Burggraf 2019, S. 569) zugunsten einer narrativen Verarbeitung in den Hintergrund treten.

Um die argumentativen und narrativen Anschlussmöglichkeiten, die in beiden Reden angelegt sind, in den kommunikativen Konstellationen der konkreten digitalen Medienumgebungen zu untersuchen, verknüpft unsere Analyse methodische Ansätze der digitalen Erzählanalyse und der Argumentationsanalyse. Ähnlich wie Page (2018) bewegt sich unsere Analyse des digitalen Erzählens auf mehreren Ebenen: von der detaillierten Analyse der jeweiligen sprachlichen Formen, mit denen gemeinsame Geschichten (›shared stories‹) erzählt und argumentativ eingesetzt werden (Mikroebene), hin zu der Frage, welche spezifischen Funktionen diese Formen des Erzählens für das Argumentieren aus kultureller Sicht haben (Makroebene).^6^ Unsere Argumentationsanalyse beruht terminologisch auf dem von Römer (2017) vorgestellten Modell der topischen Argumentationsmuster, das er im Anschluss an Klein (2000, 2002) empirisch aus der Analyse des Öl- sowie Finanzkrisendiskurses (1973/74 bzw. 2008/2009) gewinnt und das uns aufgrund dieses Krisenbezugs für den hier untersuchten Diskursausschnitt zur Corona-Pandemie als besonders geeignet erscheint. Topische Muster erfassen das »in einer Zeit verbreitete kollektive, gesellschaftliche Wissen« (Wengeler 2017, S. 268) und ermöglichen es, die in einem Diskurs vorhandenen (in der Regel impliziten) Argumentationsstrukturen offenzulegen. Römer (2017) unterscheidet folgende vier allgemeine, übergeordnete Topoi: Datentopos, Topos aus den Ursachen, Topos aus den Maximen und Finaltopos. Datentopos und Topos aus den Ursachen sind dabei mit der Conclusio wechselseitig verbunden, da es hierbei darum geht, die Folgen, die sich aus der Beschreibung der Situationsdaten und der Ursachen ergeben, mittels einer Schlussregel abzuleiten. Ebenso sind Topos aus den Maximen und der Finaltopos aufeinander bezogen, gleichzeitig liefern sie aber auch die zugrunde liegenden Wertmaßstäbe, Überzeugungen und Prinzipien, die wiederum ebenfalls die Basis der anderen argumentativen Schlussregeln bilden. Diese allgemeinen Topoi werden durch kontextspezifische Topoi inhaltlich gefüllt, die in der Analyse aus dem Datenmaterial interpretativ – und damit hermeneutisch – erschlossen und benannt werden müssen (vgl. hierzu Römer 2017; Römer/Wengeler 2013; Wengeler 2017).

## Analyse

Zentral für alle untersuchten Social-Media-Kanäle ist der Befund, dass erzählt wird, um die von Kurz und Merkel in den Lockdown-Reden aufgeworfenen Thesen und Appelle zu bestätigen oder abzulehnen. Im Folgenden analysieren wir die argumentative Funktion des Erzählens an zwei exemplarischen Bereichen, die zu den wichtigsten thematisch-funktionalen Anschlussstellen im plattformübergreifenden Kommentardiskurs gehören: An den zentralen Appell an die Vernunft der Bürger*innen schließen Kommentare an, die vom eigenen Verhalten oder der Unvernunft der anderen erzählen (4.1); an die Erzählungen vom Einsatz ›systemrelevanter‹ Berufsgruppen in den Lockdown-Reden und dem Dank seitens der Regierungsspitzen schließen Nutzer*innen-Kommentare an, die den Dank argumentativ erwidern, narrativ erweitern oder mit politischen Forderungen verbinden (4.2). Abschließend möchten wir mit einer exemplarischen Analyse eines Kommentar-Verlaufs zeigen, wie Erzählen und Argumentieren in der Social-Media-Interaktion sequenziell verbunden werden (4.3). Alle Nutzer*innen-Kommentare werden im Wortlaut und teilanonymisiert wiedergegeben.

### Anschlussstelle 1: Appell an die Vernunft und Verantwortung

Als Gemeinsamkeit über alle untersuchten Social-Media-Plattformen hinweg ist festzustellen, dass die Nutzer*innen im Zuge eines Lobs oder Danks die Notwendigkeit und Eindringlichkeit der Appelle hervorheben. Die persönliche Erlebnisperspektive, die beim Erzählen aus der Ich-Perspektive in den Vordergrund rückt, wird dabei beim Argumentieren als Ressource der Glaubwürdigkeit und Anschaulichkeit genutzt wie in den folgenden Beispielen:

1. Eine sehr bewegende Rede 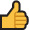 Ich finde es toll, dass erst an die Vernunft und Verantwortung jedes einzelnen appelliert wird, bevor das drastische Mittel mit sozusagen »Hausarrest« eingesetzt wird. Ich selbst bin seit 3 Wochen wegen einer simplen Erkältung zu Hause und gehe nur Einkaufen. 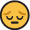 (Petra W., FB-Merkel, 18.03.2020, 59 Reaktionen/3 Antworten)

In (1) greift eine Nutzerin Merkels Appell »an die Vernunft und Verantwortung jedes einzelnen« auf und führt ihre eigene Situation, wegen einer Erkältung zu Hause zu bleiben und nur zum Einkaufen das Haus zu verlassen, als Exemplum und somit als vorbildliches und »vernünftiges« Verhalten an. Der Beleg zeigt folgende Argumentationsstruktur: Für die These (der Appell an die Eigenverantwortung ist besser als Hausarrest) wird die Erzählung des eigenen Zuhausebleibens als Argument angeführt: Weil alle (so wie ich) daheim bleiben und so zur Krisenbewältigung beitragen können, ist der Appell an die Eigenverantwortung besser als Hausarrest (Schlussregel). Dieser Topos der vorbildlichen Bürger*innen lässt sich als Topos aus den Maximen verstehen und stellt eine Ergänzung der von Merkel ins Feld geführten Topoi aus den Maximen (»solidarisches Handeln«; »was jeder und jede einzelne dazu beitragen kann«) sowie den Datentopoi (»Millionen von Ihnen können nicht zur Arbeit, Ihre Kinder können nicht zur Schule oder in die Kita, Theater und Kinos und Geschäfte sind geschlossen, und, was vielleicht das Schwerste ist: uns allen fehlen die Begegnungen, die sonst selbstverständlich sind. Natürlich ist jeder von uns in solch einer Situation voller Fragen und voller Sorgen, wie es weitergeht.«) dar und führt so den Argumentationsgang digital fort. Das Erzählen vom eigenen Verhalten kann auch als Verhaltensleitfaden, als *best practice*, verstanden und mit einem persönlichen Appell, also einer explizit formulierten Conclusio, verbunden werden, sich dem anzuschließen, wie in Beispiel 2:

(2) Ich und meine Familie machen mit seit mehr als einer Woche. Wir bleiben einfach immer zu Hause. Allein ich gehe einmal pro Woche für max. 1 Stunde einkaufen. Die Kinder können bei sonnigem Wetter im Balkon spielen. Hände, Mund, Nase und Gesicht alle ca. 2 Stunden gründlich waschen, dann gut abtrocknen. Lasst uns alle bitte mitmachen! (Khalid B., FB-Merkel, 26 Reaktionen, 0 Antworten)

Das erfahrungsbasierte Argumentieren auf der Grundlage eigener Alltagserlebnisse eignet sich dabei aber auch besonders, um Thesen der Lockdown-Reden anhand konkreter, persönlicher Eindrücke infrage zu stellen oder zurückzuweisen wie in den folgenden Beispielen (Abb. 3–4):

(3) Merkel appelliert an die Vernunft der Bürger.
  Diese Vernunft habe ich heute bei uns im Baumarkt gesehen.
  Niemand hält Abstand.
  Kunden Husten wie verrückt aber wollen sich noch bei den Blumen umschauen.
  Kunden Niesen offensichtlich in die Hand und fassen danach gemütlich Ware an.
  Großeltern gehen fröhlich mit den Enkeln Shoppen, als wäre nie was passiert. (Redacted, YT-Merkel, 656 Likes/63 Antworten)
(4) In meinen Augen verstehen es leider immer noch nicht alle in Rostock.
  Ich bin seit Montag Zuhause in Quarantäne und sehr immer noch und höre immer noch Leute wie sie draußen Corona Bier trinken und sich ein Witz daraus machen obwohl die Lage gar gar nicht mehr so witzig ist ich würde mich freuen wenn es mal endlich das was man anspricht durchgesetzt wird (Andi Anonym, YT-Merkel, 0 Likes/0 Dislikes/0 Antworten)

In (3) wird von einem Baumarktbesuch erzählt, bei dem die geltenden Hygieneregeln offenbar nicht eingehalten wurden (»Niemand hält Abstand, Kunden Husten wie verrückt [...], Kunden Niesen offensichtlich in die Hand und fassen danach gemütlich Ware an«). Diese persönliche Erzählung dient einerseits dazu, die »Vernunft der Bürger«, an die Merkel in ihrer Ansprache appelliert, infrage zu stellen, andererseits aber auch dazu, die Sinnhaftigkeit dieses Appells anzuzweifeln. Bei ersterem wird der von Merkel ins Feld geführte Topos aus den Maximen (»Appell an Vernunft der Bürger«) mit dem Topos des unvernünftigen und verantwortungslosen Bürgers kontrastiert, der zugleich sowohl den Datentopos als auch den Topos aus den Ursachen umfasst, da nicht nur ein Zustand narrativ beschrieben wird, sondern damit auch die Ursachen für etwaige Konsequenzen illustrativ vor Augen geführt werden.

Auch in (4) wird der Appell an die freiwillige Umsetzung von solidarischen Maßnahmen kritisiert. Aus einer persönlichen Vor-Ort-Perspektive wird vom Missverhalten der anderen erzählt (»sehr [= sehe, Anm. d. A.] immer und höre immer noch Leute wie sie draußen Corona Bier trinken und sich ein Witz daraus machen [...]«) und die Forderung erhoben, dass »das was man anspricht durchgesetzt wird«. Diese Forderung nach politischen Maßnahmen ergibt sich somit als Conclusio aus den persönlichen Beobachtungen, die ähnlich wie beim vorherigen Beispiel den Topos des unvernünftigen (Mit‑)Bürgers realisieren und sich auf den Datentopos sowie den Topos aus den Ursachen beziehen. Gleichzeitig wird damit erneut ein Kontrast zum Appell Merkels an die Vernunft der Bürger*innen als Topos aus den Maximen hergestellt. Ähnlich wie in (3) wird auch hier das eigene Verhalten (»Ich bin seit Montag Zuhause in Quarantäne«) als solidarisch und vorbildlich herausgestellt (= Topos des vorbildlichen Bürgers). Die Conclusio (politische Maßnahmen) ergibt sich in diesem Beispiel aus dem narrativen Kontrast zwischen dem Topos des unvernünftigen (Mit‑)Bürgers (Datentopos und Topos aus den Ursachen) einerseits sowie dem Topos des vorbildlichen Bürgers (Datentopos) andererseits. Beide Belege zeigen eine Argumentationsstruktur, in der die persönlichen Erzählungen von der ›Unvernunft der Anderen‹ die Position von Argumenten besetzen. Auf Grundlage dieser persönlichen Erlebnisperspektive wird für die These argumentiert, dass der Appell an die Vernunft (auch wenn er grundsätzlich sinnvoll sei) wirkungslos bleibt (Schlussregel: Weil die Leute – im Gegensatz zu mir – den Ernst der Lage nicht begreifen und sich nicht an die Maßnahmen halten, ist der Appell an die Eigenverantwortlichkeit wirkungslos).

Wie mit den bisher analysierten Beispielen deutlich wurde, sind es in der Regel keine ausgebauten Erzählungen, die in den Kommentaren argumentativ verwendet werden, sondern Erzählfragmente, die eine persönliche Erlebensperspektive für das Argumentieren nutzen und so als Ressource der Anschaulichkeit und Glaubwürdigkeit dienen. Diese Erzählfragmente haben dabei in der Regel den Status von Prämissen. Neben den beschriebenen fragmentarischen Wirklichkeitserzählungen, die den Status von tatsächlichen Erfahrungen behaupten, finden sich im Untersuchungsmaterial aber auch noch andere Formen des argumentativen Erzählens, die seltener auftauchen. Beispielsweise lassen sich auch hypothetische Was-wäre-wenn-Geschichten argumentativ nutzen und als Conclusio verwenden, wie in Beleg (5), in dem ein Nutzer die hypothetische Möglichkeit eines Postboten skizziert, sich und andere mit dem Coronavirus anzustecken:

(5) Können Briefe den Virus nicht relativ gut verteilen? Ich weiß nicht, wie die Lage dabei wirklich aussieht, aber wenn ein Brief von Jemandem Infizierten geschrieben wird und ein Postbote den Virus dann per Post an alle verteilt klingt das nicht ungefährlich (also, wenn der Postbote sich infiziert aber auch, wenn der Virus auf dem Brief hängt). (LLL, YT-Merkel, 0 Likes/0 Dislikes/0 Antworten)

In diesem Beispiel wird die Hypothese, dass sich Viren durch Briefe verteilen können, in Form einer Frage formuliert und mittels des hypothetischen Topos der unkontrollierten Verbreitung des Virus als Datentopos (»[…] aber wenn ein Brief von Jemandem Infizierten geschrieben wird und ein Postbote den Virus dann per Post an alle verteilt […]«) untermauert. Dies führt zu der Conclusio, dass das Briefeschreiben, zu dem die Kanzlerin in ihrer Rede aufruft (»[…] und vielleicht mal wieder Briefe schreiben. Die Post wird ja ausgeliefert.«), als »nicht ungefährlich« eingestuft und damit relativiert bzw. sogar zurückgewiesen wird. Die hypothetische narrative Sequenz dient hier – wie in einigen Beispielen zuvor – dazu, einen Kontrast zwischen Alltags- und Realitätsbezug des Kommentierenden einerseits und Realitätsferne der Kanzlerin andererseits zu etablieren. Der Appell an die Vernunft und Verantwortung geht indes nicht von der Kanzlerin aus, sondern vom Kommentar – und damit dem Kommentierenden – und zwar als indirekte Warnung und Appell, dem Aufruf der Kanzlerin nicht zu folgen.

### Anschlussstelle 2: Dank an Berufsgruppen und Helden des Alltags

An zwei prominenten Stellen ihrer Rede dankt Merkel bestimmten Berufsgruppen für ihre Arbeit während der Coronakrise: Zum einen sind dies Menschen, die als »Ärzte oder Ärztinnen, im Pflegedienst oder einer sonstigen Funktion in unseren Krankenhäusern und überhaupt im Gesundheitswesen arbeiten«. Zum anderen bezieht sich der Dank auf Menschen, die in »diesen Tagen an einer Supermarktkasse sitz[en] oder Regale befüll[en] [...] und den Laden am Laufen halten.« Solche fokussierten und bildhaft ausgestalteten Bekundungen der Dankbarkeit finden sich analog auch in der Rede von Kurz (explizit erwähnt werden die Oppositionsparteien, Beschäftigten im Gesundheitssystem, bei der Polizei, in Supermärkten und Apotheken sowie der Lebensmittelproduktion).

In vielen plattformübergreifenden Kommentaren wird dieser Dank aufgegriffen und auf weitere Berufsgruppen ausgeweitet, die nach Einschätzung der Nutzer*innen ebenfalls einen für die Krise relevanten Job ausüben. Hier zeigen sich auch plattformspezifische Besonderheiten: Insbesondere für das Erzählen im Anschluss an die Lockdown-Rede von Merkel auf YouTube ist charakteristisch, dass der Rückgriff auf den Dank an bestimmte Berufsgruppen mit der politischen Forderung nach einer finanziellen Besserstellung verbunden wird. Allerdings dienen die narrativen Episoden meist dazu, den Dank als unzureichend zurückzuweisen und stattdessen konkrete politische Forderungen aufzustellen wie in folgendem Kommentar:

(6) 12:30 Die Kassierer und Regalauffüller halten das Leben am Laufen :D Ja genau xD Unsere Bauern können ja auch mal »Zuhause« bleiben oder ich als LKW Fahrer. Wer braucht in solchen Zeiten schon Lieferungen wie Nahrungsmittel, woher bekommen Ärzte und Pfleger oder Apotheker ihre Arbeits- und Schutzmittel, die Apotheken ihre Medikamente. »Die Post kommt ja, schreiben Sie doch mal wieder einen Brief« - Ja sehr schön und die Postboten dürfen diese Briefe, die von Menschen angeleckt und angefasst werden dann austragen. Wer dankt eigentlich mal den »Putzfachkräften« die auf öffentlichen Autobahnen die Stellung halten und sich der Gefahr aussetzen, damit ich als LKW Fahrer mir nicht in die Hose scheißen muss wenn ich mal wieder 12 Stunden am Tag unterwegs bin für bisschen mehr als Mindestlohn und dann nicht mal mehr selber einkaufen kann, weil die guten Bürger des Landes alles hamstern. Aber wir müssen zusammenhalten und wir schaffen das. Einen scheiß schaffen wir. Jeder denkt wie immer nur an sich, über den Tellerrand gucken - nein danke. Aber Hauptsache es wird gedankt. Ja da kann sich die Kassiererin, das Pflegepersonal, LKW Fahrer, Putzfachkräfte, Polizisten, Feuerwehrleute, Bauern und allen anderen Alltagshelden auch nichts von kaufen. Diese Berufsgruppen halten alles zusammen, damit die Besserverdienenden Büroangestellten und vor allem Politiker etc sich weiterhin ein schönes Leben machen können. Top (Amplish, YT-Merkel, 1 Likes/0 Dislikes/0 Antworten)

In (6) gibt sich der Nutzer zunächst als LKW-Fahrer zu erkennen (»[...] ich als LKW Fahrer.«) und legt mit sehr rhetorisch aufgeladenen Worten in Form von Ironie und rhetorischen Fragen dar, weshalb seine (»Wer braucht in solchen Zeiten schon Lieferungen wie Nahrungsmittel [...]«) und andere Berufsgruppen (»Wer dankt eigentlich mal den ›Putzfachkräften‹ die auf öffentlichen Autobahnen die Stellung halten [....]«) ebenfalls krisenwichtige Aufgaben erledigen. Der Topos der Ungerechtigkeit, der dieser narrativen und emotionalen Beschreibung zugrunde liegt, bezieht sich auf den Datentopos, also die Beschreibung der aus Kommentierendensicht unhaltbaren Zustände. Die These (= These 1) lautet: Der Dank ist ungerecht verteilt, weil auch andere Berufsgruppen wie beispielsweise Apotheker*innen, Postbot*innen oder Putzfachkräfte wichtige Arbeiten verrichten (= Argument 1). Allerdings werden diese darüber hinaus ungerecht bezahlt (= Argument 2). Daraus leitet sich die Conclusio in Form von Forderungen ab, die hier ironisch und durch indirekte Sprechakte formuliert werden: Statt Dank (»Aber Hauptsache es wird gedankt.«) und Durchhalteparolen (»Aber wir müssen zusammenhalten und wir schaffen das.«) fordert er für die genannten Berufsgruppen eine bessere finanzielle Stellung (»Ja da kann sich die Kassiererin [...] auch nichts von kaufen.«) sowie mehr soziale Anerkennung, vor allem ihrer solidarischen Aufgaben (»Diese Berufsgruppen halten alles zusammen«). Beendet wird der Beitrag durch den Topos des unsolidarischen Handelns, welcher als Topos aus den Maximen eine Zuspitzung des Topos der Ungerechtigkeit darstellt: Es wird eine zweite These eingeführt (= ›bestimmte Berufsgruppen werden ausgebeutet‹), welche durch das Argument, dass sich Besserverdienende wie Büroangestellte oder Politiker auf Kosten Geringverdienender »ein schönes Leben machen können«, gestützt wird. Die Klammer zwischen These 1 und These 2 bildet hier die Conclusio, denn auch These 2 hat eine bessere soziale und finanzielle Stellung von Geringverdienern und krisenwichtigen Berufen zum Ziel. Auch in diesem Beispiel dient die Narration vor allem dazu, emotional zu argumentieren, um Topoi Nachdruck zu verleihen, oder aber aus Betroffenensicht einen emotionalen Kontrapunkt zu den Argumentationstopoi der Kanzlerin zu setzen.

In eine ähnliche Richtung geht auch die Alltagserzählung der Nutzer*in in folgendem YouTube-Kommentar (7):

(7) Schön das man zwar gelobt wird, doch oft bin ich im Einzelhandel vor dem Kunden nur der letzte Ar...
  Ich mein, es sind nicht alle so, doch von »ihr seit zu blöde zum Bestellen!« bis »Euch hat doch der Virus schon das Hirn weg gefressen!« war schon alles dabei.
  Dabei schieben wir schon Überstunden (unser Schichtplan sieht aus wie nen Weihnachtsbaum), rackern uns wund, damit die Leute nicht verhungern müssen und sind dazu noch mehr der »Seuche« ausgesetzt. Von der Bezahlung mal ganz zu schweigen.
  Wenn jeder nur etwas vernünftiger, sozialer und in Maßen einkaufen würde, hätten wir diese Engpässe garnicht.
  Das ist Paradox. Dinge bergeweise Zuhause haben wollen, damit man nicht im verseuchten Getümmel steht, aber im verseuchten Getümmel stehen um sie zu bekommen.
  Ich wünsch euch alles gute und Gesundheit. Möge das Corona Gelumpe an euch vorbei gehen. (Quei Bong, YT-Merkel, 269 Likes/0 Dislikes/35 Antworten)

Hier schildert die Nutzer*in als Arbeitskraft im Einzelhandel persönliche Negativerlebnisse, die zumeist aus Beleidigungen und Diffamierungen bestehen. Dabei stehen sich der Topos von der gesellschaftlichen Geringschätzung und der Topos der solidarischen Aufopferung als Topoi aus den Maximen gegenüber. Ersteres geht von der These aus, dass es eine Diskrepanz zwischen der Anerkennung der Kanzlerin einerseits und der sozialen Herabsetzung im Alltag andererseits gibt (»Schön das man zwar gelobt wird, doch oft bin ich im Einzelhandel vor dem Kunden nur der letzte Ar...«), was durch persönliche Erfahrungen belegt wird (»Ich mein, es sind nicht alle so, doch von ›ihr seit zu blöde zum Bestellen!‹ bis ›Euch hat doch der Virus schon das Hirn weg gefressen!‹ war schon alles dabei.«).

Der Topos der solidarischen Aufopferung beruht auf der These, dass bestimmte Berufsgruppen durch ihr Handeln die Gesellschaft zusammenhalten und auch hier dienen Alltagserlebnisse als Belege bzw. Datentopos für das Argument, dass diese Berufsgruppen ihren Beruf bis zur Selbstaufgabe ausüben, was die Brisanz und Relevanz der These auf dramatische Weise unterstreicht (»Dabei schieben wir schon Überstunden (unser Schichtplan sieht aus wie nen Weihnachtsbaum), rackern uns wund, damit die Leute nicht verhungern müssen und sind dazu noch mehr der ›Seuche‹ ausgesetzt. Von der Bezahlung mal ganz zu schweigen.«). Beide Topoi führen zur Conclusio, dass jeder durch sein solidarisches Verhalten dazu beitragen kann, die schwierige Situation zu vermeiden (»Wenn jeder nur etwas vernünftiger, sozialer und in Maßen einkaufen würde, hätten wir diese Engpässe garnicht.«). Diese ›Was wäre wenn‹-Conclusio steht allerdings im Kontrast zum Topos der selbstverschuldeten Situation als Finaltopos (»[...] hätten wir diese Engpässe garnicht.«), der die Realität abbildet, sodass die Conclusio an dieser Stelle gleichzeitig als Appell fungiert. Wie schon in vorherigen Beispielen sorgen die narrativen Episoden auch hier dafür, den Kontrast der beiden Topoi (soziale Geringschätzung im Alltag vs. solidarische Aufopferung) emotional zu betonen, zu verstärken und damit glaubwürdiger bzw. nachvollziehbarer zu gestalten.

Auch in Beispiel (7) wird die Unterbezahlung thematisiert, wenn auch nur am Rande erwähnt (»Von der Bezahlung mal ganz zu schweigen.«). Während – wie hier – in den meisten Kommentaren allgemeine Forderungen nach besserer Bezahlung erhoben werden, die auch außerhalb von Krisenzeiten – etwa in Wahlkämpfen – Gegenstand kontroverser politischer Debatten sind, gibt es auch konkrete und unmittelbare Forderungen, um bestimmten Berufen in der Krise finanziell zu helfen wie in dem folgenden Facebook-Kommentar (8):

(8) Bitte schließt die Friseure damit wir an die Hilfen kommen unsere Kunden bleiben aus, Mittel zum Schutz der Angestellten sind in unserem Handwerk schwierig, der Abstand einzuhalten wäre unmöglich. Die ersten Kollegen und Kolleginnen haben ihr Jobs schon verloren da die Inhaber der Salons leute die sich noch in probezeit oder ähnlichen Sonderregelungen befinden bereits entlassen haben. Die wenigsten dieser kleinen unternehmen haben ausreichen Rücklagen und meist hohe Mieten. (Michael P., FB-Merkel, 0 Reaktionen/0 Antworten)

Der Nutzer, offensichtlich Friseur, schildert die dramatische Lage seines Berufsstands und richtet so den emotionalen Appell an die Politik, durch Salonschließungen Zugriff auf das Coronasoforthilfeprogramm zu bekommen. Anders als in den zuvor beschriebenen Beispielen beginnt der Kommentar hier mit der Conclusio (»Bitte schließt die Friseure [...]«) sowie dem Finaltopos (»[...] damit wir an die Hilfen kommen«), gefolgt vom Topos vom wirtschaftlichen Verfall einer ganzen Branche als Datentopos (»[...] unsere Kunden bleiben aus, [...] der Abstand einzuhalten wäre unmöglich. [...] Die ersten Kollegen und Kolleginnen haben ihr Jobs schon verloren [...]«), der auch hier anhand der narrativen Schilderung konkreter Einzelbeispiele die Dramatik, Dringlichkeit und Brisanz vor Augen führt. Narrative Episoden dienen hier somit als Argumentationsstrategie, um dem Datentopos eine emotional-persuasive Wirkungsmacht und Schlagkraft zu verleihen. Auch Beispiel (9) weist ein ähnliches Argumentationsmuster auf:

(9) Als Pflegekraft auf der Intensivstation bitte ich euch im Namen aller meiner Kollegen im medizinischen Bereich: Bleibt zuhause. Lasst es nicht so kommen wie in Italien. wo momentan kriegsähnliche Zustande in der medizinischen Versorgung herrschen. Wir haben alle Angst beim Arbeiten was da auf uns zukommen wird, da wir weder genügend Beatmungsplätze und noch viel wichtiger viel zu wenig Personal haben. Bitte bitte meidet soziale Kontakte für die nächste Zeit, bleibt zuhause, denkt an die Alten aber denkt auch an uns die es ausbaden werden müssen und vielleicht nicht können…
  3.33 Der Dank an die Pflege ist übrigens schön und gut Frau Merkel, aber Taten sprechen mehr als Worte... Lasst uns den Dank doch mal z. B in angemessener Bezahlung spüren. (Milli94 × 3, YT-Merkel, 466 Likes/0 Dislikes/34 Antworten)

Der Kommentar beginnt direkt mit der Conclusio (»Bleibt zuhause«), welche als Argumentationsklammer später wieder aufgegriffen wird (»Bitte bitte meidet soziale Kontakte für die nächste Zeit, bleibt zuhause, denkt an die Alten [...]«). Zwischen dieser Klammer folgt mit dem Topos des Ausnahmezustands der Finaltopos (»Lasst es nicht so kommen wie in Italien, wo momentan kriegsähnliche Zustände in der medizinischen Versorgung herrschen.«). Die narrativen Sequenzen schließen als Datentopos (»Wir haben alle Angst beim Arbeiten was da auf uns zukommen wird, da wir weder genügend Beatmungsplätze und noch viel wichtiger viel zu wenig Personal haben.«) direkt an den Finaltopos an und dienen als Analogie-Topos dazu, die aus persönlicher Sicht und Erfahrung geschilderten Zustände den Zuständen in Italien gegenüberzustellen (wenn auch eher mahnend, da die Verhältnisse noch nicht so dramatisch sind). Durch die Sicht eines Betroffenen wird die drohende Gefahr somit personifiziert und authentisch dargestellt und lässt sich so als Konkretisierung, als illustratives Zeigen der von Merkel eher abstrakt benannten Gefahren und Appelle begreifen. Abschließend wird mit einer weiteren Conclusio abermals der Dank an die Berufsgruppe als unzureichend eingestuft und die Forderung nach »angemessener Bezahlung« erhoben. Diese Conclusio ergibt sich aber aus einem anderen Topos des Datentopos, nämlich dem Topos der Unterbezahlung, da die geschilderten Zustände aus Sicht des Kommentierenden eine angemessene Entlohnung erfordern. Somit wird deutlich, dass narrative Sequenzen mehrere Datentopoi umfassen können, um die Relevanz der in der Conclusio aufgestellten Forderungen auf illustrative, häufig dramatische, Art und Weise zu untermalen.

### Verbindung von Ko-Erzählen und Ko-Argumentieren in der Social-Media-Interaktion

Die bislang skizzierten Formen und Funktionen der narrativen Anschlusskommunikation treten mehr oder weniger ausgeprägt in allen Social-Media-Kanälen auf. Die Tatsache, dass mehrere Nutzer*innen sie plattformübergreifend aufgreifen, zeigt, dass es sich hierbei um virulente Anschlussstellen der Lockdown-Reden handelt, die Anlass zu kontroversen Anschlusskommentaren geben. Hier kann man zwei Formen des Ko-Erzählens und Ko-Argumentierens unterscheiden: Auf der übergeordneten Ebene der digitalen Medienumgebungen lassen sich ›shared stories‹ beobachten, die sich quer über mehrere Kommentare hinweg aus mehreren thematisch-funktional verwandten Anschlussäußerungen ergeben. So bilden der Appell an die Vernunft der Bürger*innen sowie der Dank an bestimmte Berufsgruppen Projektionsflächen für persönliche Erlebnisse und Eindrücke, die in vielen untereinander unverbundenen Kommentaren erzählt und argumentativ genutzt werden. Bei diesem ›horizontalen‹ Ko-Erzählen und Ko-Argumentieren ergeben sich mehrere zu einem Thema zugehörige Erzählfragmente, die gewissermaßen nebeneinanderstehen und in der Summe schlagkräftige Argumente für oder gegen etwas darstellen.^7^ Auf der Ebene einzelner Kommentarbeiträge lassen sich Ko-Konstruktionen in der Sequenz nachverfolgen. Als ›vertikale Ko-Erzähler*innen‹ gestalten Nutzer*innen hier Erzählungen sequenziell interaktiv aus. Vergleichbar zur Ko-Produktion im mündlichen Erzählen im Alltag werden hier, ausgelöst durch eine Miniatur-Erzählung, mehrere »response stories« (Tophinke 2009, 271 f.) hervorgebracht. Folgender Ausschnitt aus einer umfangreicheren Facebook-Kommentarsequenz im Anschluss an die Lockdown-Rede von Kurz bietet dafür ein anschauliches Beispiel.

(10) Na super, man will zuhause bleiben, muss aber arbeiten gehen weil nunmal Lieferdienst und Einzelhandel ausgenommen sind, was tun? Wenn dann sollte alles geschlossen werden für 2 wochen und fertig!!! (Kadisha K., Facebook-Kurz, 153 Reaktionen/51 Antworten)

Im initialen Kommentar (10) positioniert sich eine Nutzerin als Betroffene, die aus beruflichen Gründen nicht zu Hause bleiben kann, obwohl sie das gerne möchte. Damit wird an die diesbezügliche Forderung von Kurz in der Rede angeknüpft, die ungleichmäßige Arbeitsbelastung aber ohne weitere argumentative Stütze abgelehnt (»Wenn dann sollte alles geschlossen werden für 2 wochen und fertig!!!«). An diesen initialen Kommentar schließt eine Sequenz von insgesamt 51 Antworten an, in deren Verlauf sich auch die ursprünglich Kommentierende mehrmals einschaltet. In den Antworten beziehen mehrere Personen Stellung, die ebenfalls in systemrelevanten Berufen arbeiten: ein Beschäftigter in der Logistikbranche (Antwort-Kommentar 11, sowie als Nachtrag 12) und eine*r im Krankenhaus (Antwort-Kommentar 13)

(11) Kadisha K. ich arbeite selber in der logistikbranche, und bin stolz darauf das ich arbeiten gehen MUSS, denn ich weis wie viele Menschen in den nächsten wochen online bestellen werden und auf unsere hilfe angewiesen sind... das selbe gilt für die supermärkte, ein schließen kommt nicht in frage den das wäre der anfang vom ende!!! (Ermin H., Antwort-Kommentar auf 10, 210 Reaktionen)
(12) Kadisha K. und nur noch nebenbei: ich bin KEIN österreichischer staatsbürger, aber werde wenns sein muss mein leben opfern damit ich anderen helfen kann! (Ermin H., Antwort-Kommentar auf 10, 210 Reaktionen)
(13) Ich arbeite im Krankenhaus und werde weiterhin arbeiten. (Li Si, Antwort-Kommentar auf 10, 38 Reaktionen)

In diesen Antworten reagieren zwei Nutzer*innen auf den initialen Kommentar (10) und drücken durch ihre Anschlusserzählungen gegensätzliche Standpunkte aus. Das Ko-Erzählen ist hier also nicht solidarisch, sondern konfrontativ ausgerichtet: Die narrative Positionierung der kommentierenden Nutzer*innen als Beschäftigte in der Logistik (10) und in Gesundheitsberufen (»ich arbeite selber in der logistikbranche, und bin stolz darauf, das ich arbeiten gehen MUSS«, »Ich arbeite im Krankenhaus und werde weiterhin arbeiten«) ist als Hinweis auf die eigene Pflichterfüllung angelegt, die im Fall von (12) mit der Bereitschaft zur Selbstaufopferung auf die Spitze getrieben wird (»werde wenns sein muss mein Leben opfern damit ich anderen helfen kann!«). In diese Sequenz meldet sich die ursprünglich Kommentierende erneut zu Wort und elaboriert ihren initialen Post mit weiteren Details aus ihrer persönlichen Wahrnehmungswelt (14):

(14) Ermin H. ja ich arbeite auch für dpd und bin auch auf die Einnahmen angewiesen, und klar will man helfen, aber ganz ehrlich, man hat schon ein schlechtes gefühl rausgehen zu müssen obwohl von Herrn Kurz eindeutig »Heimbleiben« vorgeschrieben wird, noch dazu warum sollte der Corona Virus stopp machenvor Lidl Hofer Zustelldienste und co
  Und ganz ehrlich, Zustellung von wichtigen Medikamenten usw ist mir klar, ist wichtig!!, aber ich weiss jetzt schon dass die Leute aus Langeweile daheim, bei Zalando und co bestellen, und wir Zusteller dann vielleicht noch die Schlechte Laune der Kunden ertragen müssen 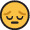 als Dank das wir in der Gefahren Zone Corona rumlaufen. 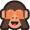 (Kadischa K., Antwort-Kommentar auf 11‑1, 31 Reaktionen)

Hier wird deutlich, dass die Konfrontation im Kommentarverlauf zu einer Argumentation für den eigenen Standpunkt führt. Die im initialen Kommentar (10) aufgestellte These (Im Lockdown sollten auch Lieferdienste geschlossen sein) wird an dieser Stelle argumentativ gestützt. Im sequenziellen Verlauf der Kommentare spielt diese Argumentation aber keine Rolle, es wird nicht mehr genauer darauf eingegangen. Vielmehr wird erneut eine deutliche Forderung an die Erst-Erzählende (15) ausgesprochen:

(15) und was sollen wir sagen, die in mobiler Dienst, in Spirale oder hauskrankepflege arbeiten? Wir machen alle unser Pflicht zu unseren Mitmenschen. Sei stolz dass du helfen kannst! (Katerina P., Antwort-Kommentar auf 11‑5, 65 Reaktionen)

Wohl nicht zuletzt aufgrund dieser massiven Konfrontation entscheidet sich die Erst-Erzählende in einem abschließenden Antwort-Kommentar (16) dafür, den Weg des kompromisslosen Dankes zu gehen:

(16) Ein Hoch auf die Ärzte und das Pflegepersonal 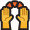 und alle die Hilfe Leisten in dieser schweren Zeit...(Kadischa K., Antwort-Kommentar auf 11, 29 Reaktionen)

Ein ganz anderer Bereich des alltäglichen Erzählens findet sich schließlich in Kommentaren, die man im Bereich von Verschwörungserzählungen verorten würde. Kommentare wie in (17) sind auf allen untersuchten Plattformen zu finden.

(17) Covid 19 Ablenkung 2020 was läuft im Hintergrund weshalb werden Überall Panzer eingeführt?? Das sollte mann sich fragen????? Ich habe angst wir werden doch nur belogen!! Und ihnen glaub ich nicht mehr (Jean Micheal, YT-Kurz, 0 Likes/0 Dislikes/0 Antworten)

In fragmentarischer Form wird hier durch das Erzählen von bestimmten Ereignissen (›Panzer im Anmarsch‹) für eine Verschwörungsthese (»wir werden doch belogen«) argumentiert. In diesen Fällen bilden die Nutzer*innen eine lose Gemeinschaft von Personen, die sich als informiert positionieren, glauben, alleine die Wahrheit zu kennen, und die Verschwörungsmythen gemeinsam teilen und nacherzählen (vgl. Weidacher 2018; vgl. zu Verschwörungserzählungen auch Niehr und Römer in diesem Band).

## Fazit und Ausblick

Ziel der qualitativen Studie war es, Muster von argumentativ verwendeten Erzählfragmenten in digitaler Anschlusskommunikation zu politischen TV-Ansprachen in Deutschland und Österreich zu untersuchen. Der Bezugstext und argumentativ-narrative Stimulus der untersuchten Social-Media-Kommentare sind die sogenannten Lockdown-Reden von Angela Merkel und Sebastian Kurz, da sie zum einen aufgrund starker emotionaler Tonalität genügend Anschlussmöglichkeiten bieten und zum anderen symptomatisch für einen aktuellen, transkulturellen Diskurs stehen.

Im Rahmen einer argumentationsbezogenen Toposanalyse wurde deutlich, dass persönliche Erzählfragmente vor allem als Datentopoi in Erscheinung treten und dabei folgende argumentative und rhetorische Funktionen erfüllen:

1. Die in den Reden aufgeworfenen Thesen und Appelle bleiben über weite Strecken abstrakt und allgemein. Die narrativen Episoden aus Sicht der Betroffenen tragen dagegen zu einer personalisierten, konkreten und damit authentischen Sicht bei. Als Glaubwürdigkeitsressource – und damit als Exemplum – stellen sie Sachverhalte und Emotionen dar und benennen sie nicht nur.
2. Sie illustrieren die Dramatik der aktuellen Zustände und erhöhen somit die Schlagkraft bzw. Dringlichkeit der in der Conclusio angeführten Konsequenzen, d. h. durch die interaktiven Koerzählungen lassen sich Argumente für (neue) politische Forderungen ableiten (z. B. bessere Bezahlung von systemrelevanten Berufen). In diesem Fall können mit (fragmentarischen) Alltagserzählungen die Thesen und Appelle in den Reden also entweder bekräftigt oder zurückgewiesen werden. Ersteres führt in vielen Fällen zu einem Kontrast zwischen Anspruch/Idealismus und Wirklichkeit sowie zwischen Realitätsferne und -fremde und damit zwischen ›denen da oben‹ auf der einen und ›uns‹ auf der anderen Seite.
3. Aus Rezipient*innensicht ermöglicht das Erzählen in Anschlusskommentaren die argumentative (Neu‑)Positionierung innerhalb des Diskurses, häufig im interaktiven Abgleich mit anderen Positionen. So ergibt sich ein aggregiertes diskursives Stimmungsbild als Ergebnis des Aneignungsprozesses.

Allerdings zeigt sich, dass eine Zuordnung zu bestimmten Topoi alles andere als eindeutig und trennscharf ist, denn in vielen Fällen lassen sich kontextspezifische Topoi unterschiedlichen übergeordneten Topoi gleichzeitig zuordnen. So realisiert z. B. der Topos des vorbildlichen Bürgers sowohl den Datentopos als auch den Topos aus den Maximen, weil die Vorbildfunktion durch ganz konkrete Handlungen narrativ dargestellt und mithin mit einem bestimmten Hochwertkonzept – nämlich *Vorbild* – assoziiert wird. Zudem wird dieses Konzept nicht an der sprachlichen Oberfläche, also durch die Nennung des Begriffs *Vorbild*, realisiert, sondern muss inferiert werden. Gerade hier scheint der ›Mehrwert‹ einer Argumentation mittels Erzählfragmenten zu liegen: Durch die emotionale Schilderung lassen sich Zustände (Daten) einerseits sowie zugrunde liegende Prinzipien, Werte und Konzepte andererseits gleichzeitig übermitteln, wobei letztere häufig implizit bleiben, also als zugrunde liegend vorausgesetzt werden. Dadurch scheint es einfacher möglich, einen Wertekontrast zwischen solidarisch – unsolidarisch, vorbildlich – nicht vorbildlich, gerecht – ungerecht, vernünftig – unvernünftig etc. zu etablieren, was auch eine soziale Abgrenzung zu Andershandelnden und -denkenden erlaubt.

Inwiefern lassen sich daraus weitergehende Schlussfolgerungen für den interkulturellen Vergleich zwischen Österreich und Deutschland ziehen? Eine detaillierte Kontrastierung der politischen Kultur in beiden Ländern auf Grundlage unserer Daten allein ist nicht möglich. Deutlich wurde jedoch, dass sich die Unterschiede, die in der Art der Rede und deren Präsentation angelegt sind, auch auf die Form und die Intensität des Erzählens in der Anschlusskommunikation auswirken. Die Rede von Angela Merkel wurde lange erwartet und so zu einem Medienereignis, das entsprechend viel Aufmerksamkeit auf sich versammeln konnte, die sich auch bei der narrativen Weiterverbreitung der Rede auf den hier untersuchten Social-Media-Plattformen wiederfindet. Die Rede von Sebastian Kurz hingegen steht im Zusammenhang mit einer Vielzahl von verschiedenen Ankündigungen, Pressestatements, Parlamentsreden, Live-Interviews etc., bei denen Aussagen von Kurz über einen längeren Zeitraum und über verschiedene Wege medial verteilt veröffentlicht wurden. Diese unterschiedliche gesellschaftliche Wahrnehmung der Rede zeigt sich auch darin, dass Merkels Ansprache relativ bald als »historisch« bezeichnet wurde (und beispielsweise auch einen eigenen Wikipedia-Eintrag hat), während die Fernsehansprache von Kurz keine solchen Spuren hinterlassen hat. Das digitale Anschlusserzählen kann man daher im Fall von Merkel in punktuell verdichteter Form auf den diversen Plattformen verfolgen, während bei Kurz die Anschlusskommunikation zeitlich und räumlich verteilt wird.

Insgesamt unterscheiden sich die narrativen Muster der deutschen und österreichischen Anschlusskommentare kaum voneinander: Übergreifend dienen bestimmte Sequenzen der Reden als Auslöser für Erzählungen, die häufig in komplexe Argumentationen eingebettet sind. Was wir in unserer Analyse nicht gut zeigen konnten, sind plattformspezifische Unterschiede. Ein Grund dafür liegt in der Heterogenität der Daten, die auf diversen Social-Media-Accounts veröffentlich wurden und im Ländervergleich zudem zum Teil deutliche Unterschiede in der Nutzungsintensität aufweisen, was einen systematischen Vergleich erschwert. Mit aller Vorsicht können wir aber abschließend auf einige Unterschiede verweisen: Wie in den Analysen bereits erwähnt, ist es für Facebook-Daten charakteristisch, dass die Reden gelobt werden und die Notwendigkeit und Eindringlichkeit der Appelle hervorgehoben werden, während auf YouTube zum Teil deutlich längere und kritischere Kommentare zu finden sind. Für die Kommentare auf Instagram scheint es insgesamt charakteristisch zu sein, dass sie sehr personenzentriert sind und fast ausnahmslos um die Außenwirkung von Kurz und Merkel kreisen. Dabei positionieren sich die Nutzer*innen entweder als Anhänger*innen oder Gegner*innen der Politiker*innen, dazwischen scheint es nichts zu geben. Diese Polarisierung wirkt sich insofern auf das Erzählen aus, als themenbezogene Argumentationen und längere Narrationen auf Instagram nur äußerst selten zu finden sind. Die größten Unterschiede im Vergleich zu den anderen Plattformen lassen sich für Twitter festhalten. Hier ist ebenso eine starke Polarisierung zwischen Anhänger*innen und Gegner*innen zu beobachten, allerdings ist diese deutlich (partei‑)politisch motiviert (vgl. hierzu auch Klemm/Michel 2016). Für die Gruppe der Anhänger*innen ist charakteristisch, dass sie – ähnlich wie bei Instagram – die Lockdown-Reden lobt und die dort hervorgebrachten Argumente und Appelle unterstützt, häufig verbunden mit dem Eindringlichkeitstopos wie er vor allem bei Facebook gehäuft auftritt. Eine Besonderheit des Erzählens auf Twitter ist, dass sich hier auch zahlreiche Spitzenpolitiker*innen an der metakommunikativen Kommentierung der Lockdown-Reden beteiligen und die Reden auch im Kontrast mit den Ansprachen anderer Staatschefs (Macron, Trump etc.) vergleichen. Insgesamt bleibt aber festzuhalten, dass narrative Sequenzen bei Twitter – bezogen auf unser Korpus – die Ausnahme darstellen.

Es wäre an weiteren politischen TV-Ansprachen – auch zu unterschiedlichen Diskursen – zu überprüfen, inwiefern es sich hier tatsächlich um universelle Muster von Argumentation mittels Narration handelt, oder ob es kulturspezifische Ausprägungen gibt. Ferner, ob sich weitere narrative Muster erkennen lassen und wie sie gegebenenfalls zu typologisieren sind. Gerade bei digitalem Erzählen kommt der mediendispositiven Gestaltung eine wichtige Bedeutung zu, sodass in einem nächsten Schritt die hier nur tentativ und tendenziell – an einem kleinen Korpus – gewonnenen Beobachtungen zu der Plattformspezifik narrativer und argumentativer Muster anhand umfangreicherer Datenmengen verifiziert werden sollten. Schließlich wäre das Offenlegen der Mechanismen des gemeinschaftlichen Erzählens von Verschwörungsmythen, das sich auch in unseren Daten an mehreren Stellen zeigt, eine wichtige Aufgabe einer (medien-)linguistischen Analyse des vernetzten Erzählens im Kontext mediatisierter Politik.

## Footnotes

1 Wir bedanken uns bei den Herausgeberinnen für die hilfreichen Anregungen zur ersten Fassung des Manuskripts.

2 Nach Hickethier (1995) ist das Dispositiv Fernsehen – und damit die Besonderheiten der Fernsehnutzung und Rezeptionserfahrung des Mediums – durch eine spezifische Anordnung von Rezipierenden, technischen Geräten und institutionellen ›Apparaten‹ geprägt. Im Anschluss an den Dispositivbegriff von Foucault (1978) sei Fernsehkommunikation durch diese größeren Rahmen, die von den einzelnen Zuschauer*innen nur indirekt und nicht bewusst erlebt oder wahrgenommenen werden, auch im Zusammenhang mit Machtaspekten analysierbar (vgl. Hickethier 1995, S. 69).

3 Als ›kommunikative Mediendispositive‹ bestimmen Social-Media-Plattformen den strukturellen, sozialen, interaktiven und funktionalen Kommunikationsrahmen, was wiederum Einfluss auf die Ausgestaltung der jeweiligen dort vorherrschenden Kommunikationsformen- und Textualitätsaffordanzen hat (vgl. Michel 2018).

4 Ein solches bildschirmbasiertes Verfahren (vgl. Androutsopoulos 2018) eignet sich in erster Linie für die Beiträge, die von Nutzer*innen produziert werden, berücksichtigt aber auch die kontextuelle und multimodale Einbettung in die Medienumgebung. In einem ersten Schritt haben wir jeweils die ersten 80 Kommentare erhoben und mit Metadaten (u.a. Zeitstempel, Anzahl der Favorisierungen, Teilungen und Antwort-Kommentare) dokumentiert. Bei Facebook, Instagram und YouTube haben wir uns grundsätzlich in einem ersten Schritt auf die Top-Level-Kommentare beschränkt, aber auf Grundlage einer Vor-Analyse auch längere Interaktionsverläufe punktuell berücksichtigt.

5 Emotionalisierung verstehen wir hier als übergreifende Strategie, die verschiedene sprachliche Mittel und Verfahren nutzen kann. Im Fall der Reden zählt dazu u.a. ein expressiver und Emotionen thematisierender Sprachgebrauch. Während Emotionsausdruck durch sprachliche Elemente, d.h. objektsprachlich, realisiert ist, wird bei Emotionsbenennung auf Emotionen metasprachlich referiert. So wird die Darstellung der dramatischen Situation in Italien in der Lockdown-Rede von Kurz unter der Benennung von Emotionen (in diesem Fall: Angst) metakommunikativ evaluiert: »Und ich sage das nicht, um Ihnen Angst zu machen, sondern ich sage es, weil es die Wahrheit ist, und weil es noch immer zu viele Menschen in unserem Land gibt, die versuchen zu beschwichtigen und die Dinge schönreden.« (Lockdown-Rede Sebastian Kurz, 15.03.2020). Emotionalisierung verbindet sich dabei auch mit anderen Strategien, wie beispielsweise einer Personifizierung. So forciert Merkel in ihrer Rede eine Identifikation der Zuschauer*innen mit den Coronaopfern und den Betroffenen: »Das sind nicht einfach abstrakte Zahlen in einer Statistik, sondern das ist ein Vater oder Großvater, eine Mutter oder Großmutter, eine Partnerin oder Partner, es sind Menschen.«.

6 Dazwischen lässt sich mit Page (2018) noch eine Ebene verorten, die auf die technologischen Affordanzen fokussiert. Hier geht es um die Frage, wie digitales Erzählen und Argumentieren durch verschiedene technologische Ressourcen, die in den Produktions- und Rezeptionskontexten eingesetzt werden, vermittelt werden. Diese medienlinguistisch interessante Mesoebene können wir in der Analyse nur am Rande behandeln (vgl. dazu die abschließende Diskussion in Abschnitt 5).

7 Der Zusammenhang von Argumentieren und den medialen Rahmenbedingungen wird damit jedoch erst bruchstückhaft behandelt. So müsste beispielsweise die quantitative Dimension des Argumentierens auf Social-Media-Plattformen näher aufgeführt werden. Neben der Tatsache, dass mehrere ähnliche Argumente zusammen ein Mehrheitsargument bilden (wenn eine große Anzahl von Menschen X so sieht, dann muss X wahr/richtig sein), geht es dabei auch um technische Rahmenbedingungen. Das betrifft etwa die flächige Sichtbarkeit der Social-Media-Interaktion. Zum Beispiel können mehrere ähnliche Argumente durch den Platz, den sie auf dem Bildschirm einnehmen, eine Art ikonisches Argument bilden (wenn die Argumente für X so viel Platz einnehmen, dann muss X wahr/richtig sein).
